# Transcriptomic analysis identifies candidate genes for Aphanomyces root rot disease resistance in pea

**DOI:** 10.1186/s12870-024-04817-y

**Published:** 2024-02-28

**Authors:** Carol Kälin, Edoardo Piombo, Salim Bourras, Agnese Kolodinska Brantestam, Mukesh Dubey, Malin Elfstrand, Magnus Karlsson

**Affiliations:** 1 Department of Forest Mycology and Plant Pathology, Swedish University of Agricultural Sciences, Uppsala, Sweden; 2 Nomad Foods Ltd, Findus Sverige AB, Bjuv, Sweden

**Keywords:** Abscisic acid, *Aphanomyces euteiches*, Candidate disease resistance genes, Differential gene expression, Immune receptor, Pea breeding, *Pisum sativum*, Transcriptomics

## Abstract

**Background:**

*Aphanomyces euteiches* is a soil-borne oomycete that causes root rot in pea and other legume species. Symptoms of Aphanomyces root rot (ARR) include root discoloration and wilting, leading to significant yield losses in pea production. Resistance to ARR is known to be polygenic but the roles of single genes in the pea immune response are still poorly understood. This study uses transcriptomics to elucidate the immune response of two pea genotypes varying in their levels of resistance to *A. euteiches*.

**Results:**

In this study, we inoculated roots of the pea (*P. sativum* L.*)* genotypes ‘Linnea’ (susceptible) and ‘PI180693’ (resistant) with two different *A. euteiches* strains varying in levels of virulence. The roots were harvested at 6 h post-inoculation (hpi), 20 hpi and 48 hpi, followed by differential gene expression analysis. Our results showed a time- and genotype-dependent immune response towards *A. euteiches* infection, involving several WRKY and MYB-like transcription factors, along with genes associated with jasmonic acid (JA) and abscisic acid (ABA) signaling. By cross-referencing with genes segregating with partial resistance to ARR, we identified 39 candidate disease resistance genes at the later stage of infection. Among the genes solely upregulated in the resistant genotype ‘PI180693’, Psat7g091800.1 was polymorphic between the pea genotypes and encoded a Leucine-rich repeat receptor-like kinase reminiscent of the *Arabidopsis thaliana* FLAGELLIN-SENSITIVE 2 receptor.

**Conclusions:**

This study provides new insights into the gene expression dynamics controlling the immune response of resistant and susceptible pea genotypes to *A. euteiches* infection. We present a set of 39 candidate disease resistance genes for ARR in pea, including the putative immune receptor Psat7g091800.1, for future functional validation.

**Supplementary Information:**

The online version contains supplementary material available at 10.1186/s12870-024-04817-y.

## Background

Green pea (*Pisum sativum* L.) belongs to the Fabaceae family (or Leguminosae), and is cultivated worldwide in cool temperate areas [1]. The legume poses a valuable source of plant-based protein for food and feed [2], and the global production has been increasing steadily [3]. However, pea cultivation faces several biotic and abiotic constraints, most notably soil-borne pathogens causing root rot [4, 5]. Root rot in pea is caused by a complex of fungal and oomycete pathogens, whereas Aphanomyces root rot (ARR) is the most devastating threat to pea production in main vining pea production areas with temperate climate [6, 7].

The causative agent of ARR is *Aphanomyces euteiches,* which is a homothallic (self-fertile) oomycete with a broad host range on various legume species. The pathogen has a hemibiotrophic lifestyle, completing a shift from a biotrophic to necrotrophic growth phase on its host plant. An infection cycle starts with oospore germination and the production of asexual bi-flagellate motile zoospores, which detect root exudates and continue to encyst and penetrate the root system [7, 8]. In the first six days of infection, the biotrophic phase, the pathogen colonizes the cortex root tissue of the host plant. The necrotrophic growth phase is initiated by the invasion of the stele and vascular tissues, leading to the typical browning of the roots and premature plant death [9, 10]. The cycle ends with the production of sexual oospores in declining host tissues [11]. Oospores are particularly problematic in pea cultivation, as they can remain resilient in the soil for a long time [12]. Long periods of crop rotation and avoidance of highly infested fields are often the only effective measures in the mitigation of ARR [13, 14]. Understanding the molecular basis of host resistance in pea to ARR and the integration of resistant pea varieties would be the economically and ecologically most beneficial strategy in the mitigation of ARR.

There is currently no commercial pea variety with complete resistance to ARR, but the landrace ‘PI180693’ has been used as a source of resistance in commercial breeding programs [15]. However, ‘PI180693’ is unsuitable for commercial cultivation due to poor green pea quality (pale seed coat color, mealy and hard texture, lack of sweetness) as well as agronomic properties unfit for modern large scale crop cultivation (e.g. long internodes, susceptibility for powdery and downy mildew). The pea cultivar ‘Linnea’ on the other hand, bears favorable agronomic and green pea quality traits and has been used in commercial production in Sweden since 2010. However, ‘Linnea’ is highly susceptible to ARR. The levels of susceptibility of both pea genotypes to ARR have previously been evaluated in the field, and controlled greenhouse trials [16, 17].

The *P. sativum* genome is among the largest in legumes as its haploid size corresponds to 4.45 Gb on seven paired chromosomes. For the first annotated chromosome-level assembly for *P. sativum*, the French cultivar ‘Caméor’ was sequenced by Kreplak et al. [18] and has since been facilitating the development of genetic markers. Resistance to ARR in pea is quantitative and polygenic. Several consistent Quantitative Trait Loci (QTL) associated with partial resistance to ARR have been identified and validated in pea, paving the way for marker-assisted selection in breeding programs [19–25]. A cross between a susceptible and resistant pea cultivar was used to identify QTL for partial resistance to ARR based on greenhouse and field experiments, and ultimately identified the gene content in the ARR resistance QTL [26]. Genes segregating with ARR resistance were further expanded using bulked segregant RNA-seq (BSR-seq) analysis and used for cross-referencing with differentially expressed genes (DEGs) [27].

The use of transcriptomics in controlled host–pathogen infections allows the identification of candidate disease resistance genes and has been employed successfully in the field of legume-microbe interactions [28, 29]. In many studies, the legume model species *Medicago truncatula* is used to study the immune response towards *A. euteiches*. Badis et al. [30] for example, used a transcriptomics approach to identify genes involved in defence and signaling pathways that are associated with partial resistance to *A. euteiches* in *M. truncatula*. Hosseini et al. [31] investigated the transcriptional immune response in pea towards two oomycete pathogens, *Phytophtora pisi* and *A. euteiches*, and identified chalcone synthases and genes active in the auxin pathway to be specifically upregulated upon *A. euteiches* infection. Williamson-Benavides et al. [32] identified induced immune response genes in a susceptible *P. sativum* host upon infection with *Fusarium solani* f. sp. *pisi* compared to a partially resistant host. However, limited information is available about the genetic interaction between *A. euteiches* and the resistance level of its pea host during infection or how varying levels of *A. euteiches* virulence affects the pea immune response. Although *A. euteiches* strains are assigned to races based on their pathogenicity against alfalfa cultivars [33], little is known about how the transcriptomic immune response in their respective host is affected.

In the current study, we performed a transcriptomic analysis of two different pea genotypes with varying levels of ARR resistance, upon infection with two different *A. euteiches* strains with varying levels of virulence. Virulence was defined as the severity of disease symptoms after inoculation with *A. euteiches*. We hypothesized that i) partial resistance towards ARR is associated with different sets of DEGs in the susceptible and resistant pea cultivar, ii) genes that are differentially regulated upon *A. euteiches* infection are preferentially located in ARR resistance QTL, and that iii) there is an *A. euteiches* virulence-dependent transcriptional response in the two pea genotypes upon infection.

## Results

### Immune response in pea is determined by quantitative resistance in the host rather than the virulence level of *A. euteiches*

**Fig. 1:**
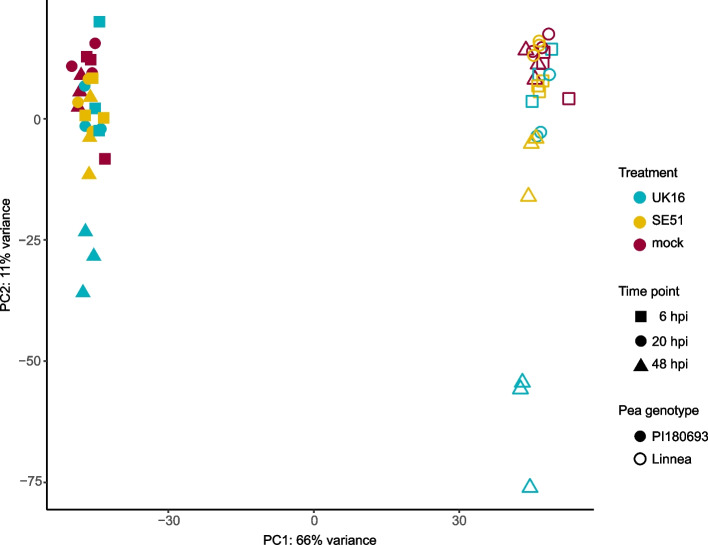
Principal component analysis (PCA) of the transcriptomics data set including three biological replicates for the *A. euteiches* treatments (highly virulent UK16 and lowly virulent SE51) and mock control, root harvesting time points (shapes) and the pea genotypes ‘PI180693’ (filled shapes) and ‘Linnea’ (empty shapes)

Seedlings of ‘Linnea’ and ‘PI180693’ were inoculated by dipping into a zoospore solution of *A. euteiches* strains UK16 or SE51, consistently shown to differ in virulence on ‘Linnea’ and ‘PI180693’ in climate chamber trials [17]. The ‘Linnea’ seedlings serving as infection control were left in the open pipette boxes for several days and confirmed successful disease development by visual inspection in seedlings treated with *A. euteiches* strains UK16 and SE51, and the absence of disease symptoms in the mock treatments. The average number of million reads per sample ranged from 47.1 to 77.6, representing sufficient amount of sequence data for analyzing differential gene expression (Table S2). Principal Component Analysis (PCA) of the entire dataset showed a clear clustering according to pea genotypes, but not to treatment with *A. euteiches* strains (Fig. 1). Further, PCAs split by pea genotype showed a separation by time point but no clear separation by *A. euteiches* virulence levels, except for ‘PI180693’ at 48 h post-inoculation (hpi), inoculated with the more virulent UK16 (Figure S2).

### Exponential increase in *A. euteiches* biomass and DEGs in ‘Linnea’ upon infection with strain UK16

**Fig. 2:**
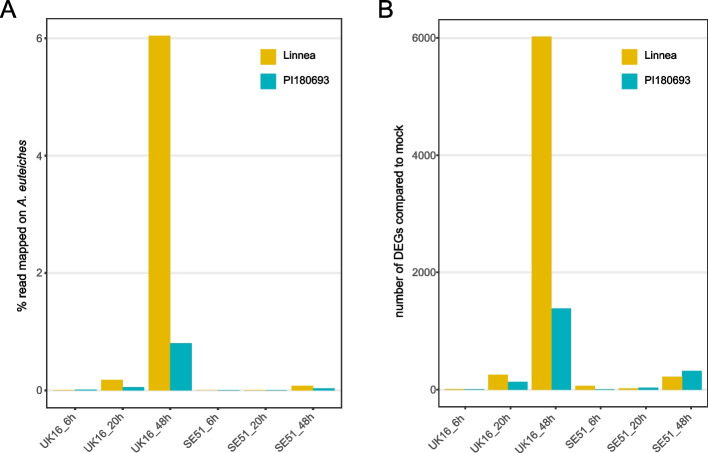
**A** The percentage of reads that mapped on the *A. euteiches* reference genome for the pea genotypes ‘Linnea’ (yellow) and ‘PI180693’ (blue) for every *A. euteiches* treatment (highly virulent strain UK16 and lowly virulent SE51) and time point. **B** Increasing number of differentially expressed genes (DEGs) with absolute value of log2FC > 1 and adjusted *p*-value < 0.05, compared to mock treatment for ‘Linnea’ (yellow) and ‘PI180693’ (blue), separated by *A. euteiches* treatment and time point

To confirm an increasing presence of *A. euteiches* biomass during the infection process, we assessed the percentage of reads that mapped to the *A. euteiches* reference genome, as a proxy for biomass. The highest percentage of reads mapping to the *A. euteiches* genome was observed at 48 hpi in ‘Linnea’ upon inoculation with the highly virulent strain UK16. For all time points, more *A. euteiches* reads mapped in interaction with the susceptible pea genotype compared to the partially resistant ‘PI180693’ and strain UK16 accounted for more biomass in all conditions (Fig. 2a). This difference was most apparent at time point 48 hpi, where 9.5 times more reads were assigned to *A. euteiches* when infecting ‘Linnea’ as compared to ‘PI180693’ (Table S2). We observed low numbers of differentially expressed genes (DEGs, absolute value of log2FC > 1) at the early time points 6 hpi and 20 hpi with either *A. euteiches* strains and in both pea genotypes (Fig. 2b). Most DEGs were scored in ‘Linnea’ upon infection with the more virulent strain UK16 at 48 hpi. At the same time point, and at 20 hpi, we observed more DEGs in ‘PI180693’ compared to ‘Linnea’ upon infection with strain SE51. Overall, numbers of DEGs as well as *A. euteiches* reads were increasing with time and higher in the treatments with the highly virulent strain UK16. Numbers of DEGs at all time points and conditions, as well as normalized read counts are listed in Table S3.

### The transcriptional immune response of pea to *A. euteiches* is time-dependent

We identified 75 DEGs at 6 hpi and 375 DEGs at 20 hpi (Table S4) and retrieved the available information of the corresponding genes from the pea genome database (https://urgi.versailles.inra.fr/download/pea/), including gene ontology (GO) terms for all genes (Tables S5 and S6). At the earliest time point, we identified three seed linoleate 9S-lipoxygenase-3-like genes that were previously associated with partial resistance to Aphanomyces root rot (ARR) and predicted to be involved in oxidation–reduction processes and jasmonic acid (JA) biosynthesis [27]. Additionally, Psat2g149200.1, Psat5g289880.1 and Psat5g291320.1 were all downregulated in ‘Linnea’ upon infection with SE51 (Table S5).

At 20 hpi, more genes associated with the GO term “defense response to other organisms” (GO:0009814) were upregulated in ‘Linnea’ (eleven) than in ‘PI180693’ (four). A similar pattern was observed for predicted receptor-like kinases, where 17 were upregulated in ‘Linnea’, two of which were also upregulated in ‘PI180693’. We identified seven genes putatively involved in disease resistance responses to be upregulated at 20 hpi. Disease resistance response proteins Pi176 and Pi49 have GO terms connected to abscisic acid (ABA) binding and were both upregulated in ‘PI180693’ but not in ‘Linnea’. Psat2g115400.1 was upregulated in both pea genotypes, while Psat2g013480.1, Psat7g028600.1, Psat7g029960.1 and Psat7g028560.1 were upregulated only in ‘Linnea’.

Among other upregulated genes in ‘Linnea’ we found ethylene-responsive transcription factors (TFs, Psat6g137360.1, Psat6g054800.1), an auxin-responsive, as well as ABA-responsive ABR18-like gene (Psat7g037160.1 and Psat6g217920.1). Additionally, we found four myeloblastosis (MYB)-like and six WRKY TFs (Table S5). Two chitinases (Psat1g150520.1, Psat1g148600.1) were among downregulated genes in ‘Linnea’ at 20 hpi. In ‘PI180693’, we found TFs *myb14*-like and *myb15*-like genes (Psat6g137320.1 and Psat6g105240.1) and gene Psat1g157240.1, encoding the disease resistance response protein Pi176, among the most upregulated DEGs. Upon infection of ‘PI180693’ with either *A. euteiches* strain, we found TF *myb102* (Psat1g209120.1) and abscisic acid and environmental stress-inducible protein encoding gene Psat2g026840.1 to be downregulated (Table S5).

Five of the differentially regulated genes at 20 hpi were located in genomic regions segregating with partial resistance to ARR. Psat4g140440.1, a probable leucine-rich repeat (LRR) receptor-like serine/threonine-protein kinase and Psat7g083880.1, a leaf rust 10 disease resistance locus receptor-like protein kinase homolog [26], were both upregulated in ‘Linnea’ upon infection with strain UK16. The other three genes were associated with hormone metabolism where Psat3g026920.1 was predicted to be part of methylsalicylate degradation. Genes Psat5g289880.1 and Psat5g291320.1 were associated with oxidation–reduction processes and JA biosynthesis and were among the most downregulated genes in ‘PI180693’ [27] (Table S5).

### Specific immune response differing between pea genotypes becomes apparent with progressing *A. euteiches* infection

**Fig. 3:**
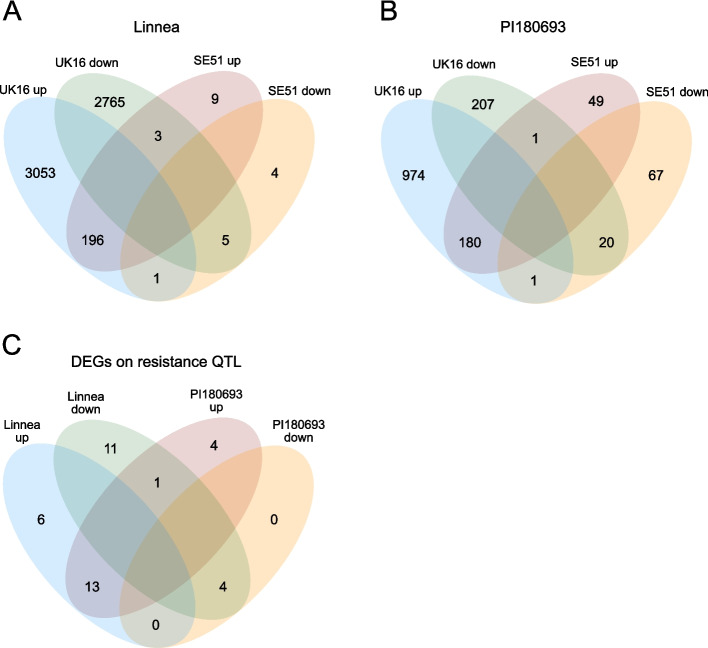
Differentially expressed genes (DEGs, absolute value of log2FC > 1 and adjusted *p*-value < 0.05, compared to mock treatment) in the susceptible pea genotype ‘Linnea’ (**A**) and resistant ‘PI180693’ (**B**), split by *A. euteiches* strains UK16 (high virulence) and SE51 (low virulence) and up- and downregulation. **C** Non-strain specific DEGs segregating with loci for partial resistance to Aphanomyces root rot as previously described by Wu et al. 2021 and 2022

At the later stage of infection, 48 hpi, we identified a total of 6036 DEGs in ‘Linnea’ and 1499 DEGs in ‘PI180693’ (Tables S3 and S4). At 48 hpi, we counted considerably more DEGs in both pea genotypes upon infection with the highly virulent strain UK16 than with strain SE51 (Figs. 3A, B). In ‘Linnea’, 196 DEGs were upregulated upon infection with either *A. euteiches* strain, comprising the majority (94.2%) of upregulated genes in the interaction of ‘Linnea’ and SE51 (Fig. 3A). In ‘PI180693’, 180 DEGs were upregulated in a non-strain specific manner, which accounted for 78.3% of genes upregulated upon infection with SE51 and only 15.6% of genes upregulated upon infection with UK16 (Fig. 3B).

In response to the more virulent strain UK16, the susceptible genotype ‘Linnea’ displayed more DEGs enriched (*p* < 0.05) for GO terms “defense response” than the resistant ‘PI180693’. In response to the less virulent strain SE51, GO terms associated with upregulated DEGs in both ‘Linnea’ and ‘PI180693’ comprise “responses to biotic stimuli”, “(protein) phosphorylation”, and in ‘PI180693’ specifically “responses to (oxidative) stress” (Table S6).

Due to the great number of DEGs at 48 hpi, we focused on the 25 most strongly regulated genes upon infection in both pea genotypes for every condition and Table 1 shows a selection of genes with predicted defense-related gene functions and their closest characterized homolog. In general, interactions involving UK16 but not SE51 were very frequent among these strongly DEGs including a strongly downregulated seed linoleate 9S-lipoxygenase-3-like gene, Psat4g185080.1 (Table 1). Two more seed linoleate 9S-lipoxygenase-3-like genes, Psat5g289880.1 and Psat5g291320.1, were found among the strongly DEGs (Table 1). The allene oxide synthase 1 gene (Psat0s2724g0160.1) was induced in the interactions between ‘Linnea’ and UK16 and ‘PI180693’ and the low virulent strain SE51, but no significant induction was observed in the other two conditions (Table S5).

**Table 1:** Differentially expressed genes among 25 most up- and downregulated genes in ‘Linnea’ and ‘PI180693’ at 48 hpi associated with predicted defense response

Gene id	Closest BLAST hit	Linnea UK16	Linnea SE51	PI180693 UK16	PI180693 SE51
Psat0s1622g0080.1	NDR1/HIN1-like protein 10	2.501	n.s	n.s	-2.073
Psat2g109600.1	MLP-like protein 423	-3.685	n.s	-2.466	n.s
Psat4g185080.1*	Oxidation–reduction process, 9S-lipoxygenase-3-like	-6.734	n.s	-2.567	n.s
Psat4g182840.1	Disease resistance protein RPV1-like	4.218	1.175	2.261	n.s
Psat5g289880.1*	Oxidation–reduction process, 9S-lipoxygenase-3-like	-1.507	n.s	n.s	5.411
Psat5g242640.1	Pathogenesis-related protein	2.412	n.s	2.839	5.576
Psat5g291320.1*	Oxidation–reduction process, 9S-lipoxygenase-3-like	n.s	n.s	n.s	5.741
Psat6g146200.1	Pathogenesis-related protein PR-4-like	9.274	5.081	10.142	6.400
Psat6g109120.1	Pathogenesis-related protein PR-4-like	8.936	n.s	7.875	n.s
Psat6g042680.1	MLP-like protein 34	n.s	n.s	-2.448	n.s
Psat7g035720.1	Putative thaumatin	8.733	4.801	8.258	5.198
Psat7g029960.1	Disease resistance response protein 206-like	8.082	n.s	7.144	5.338
Psat7g036280.1	Thaumatin-like protein	8.585	4.371	8.915	5.439

^*^Genes segregating with partial resistance to Aphanomyces root rot described in Wu et al. 2022, *n.s*. Non-significant, differential gene expression compared to mock treatments, absolute value of log2FC > 1, adjusted *p*-value < 0.05

Fourteen genes were among the most upregulated genes across all interactions (Table S5). This group included several genes with similarity to known PR-protein genes (e.g. Psat1g211480.1, Psat6g146200.1, Psat7g035720.1 and Psat7g036280.1). An interesting set of genes in this analysis consisted of 24 genes that were highly upregulated in all interactions, except between ‘PI180693’ and the less virulent strain SE51. Among these genes were three transcription factor genes, two encoding WRKY transcription factors (Psat6g026680.1 and Psat5g236440.1) and one gene with similarity to the *rax3* MYB transcription factor (Psat4g080720.1) (Table S5). Eleven genes were strongly differentially regulated in all interactions except for the interaction between ‘Linnea’ and SE51, where no significant difference was found. This group of genes frequently lacked similarity with characterized genes but the strongly upregulated Psat6g137320.1 was similar to *myb14* transcription factors, while Psat1g001480.1 was upregulated in the interaction between ‘PI180693’ and the less virulent strain SE51 and was similar to 9-cis-epoxycarotenoid dioxygenase* nced1* (Table S5).

### Thirty-nine candidate disease resistance genes at 48 hpi were previously associated with partial resistance to ARR

Differentially regulated genes at 48 hpi were cross-referenced with genes localized in genomic regions segregating with ARR resistance [26, 27]. The 39 genes displayed in Fig. 3C and Table 2 represent the non-strain specific immune response of ‘Linnea’ and ‘PI180693’. Among the genes upregulated only in ‘Linnea’, Psat1g156920.1, encoding an ABR17-like protein, and Psat4g025040.1, a possible nodulin-13-like protein, were associated with the ABA-activated signaling pathway. Downregulated DEGs in the quantitative trait locus (QTL) specific to the susceptible ‘Linnea’ comprised three major latex protein (MLP)-like genes, two genes encoding disease resistance proteins (RFL1-like and RPM1-like), as well as two LRR receptor-like tyrosine protein kinase genes (Table S5). Upregulated genes in the QTL in both ‘Linnea’ and ‘PI180693’ involved two receptor-like protein kinases, Psat4g140440.1 and Psat6g203640.1. Among the downregulated genes associated with the QTL regions in both pea genotypes were three genes associated to oxylipin biosynthesis, Psat4g184760.1, Psat4g185080.1 and Psat5g289880.1. Interestingly, four DEGs associated with the QTL regions were upregulated exclusively in ‘PI180693’ at 48 hpi in response to *A. euteiches* infection. These include Psat2g013520.1, a predicted resistance to *Uncinula necator* 1 (RUN1)-like disease resistance protein, involved in signal transduction and originally described in the grapevine species *Muscadinia rotundifolia* for its resistance to powdery mildew [34, 35]. The second gene, Psat5g242600.1, a predicted *P. sativum* defensin 2 (Psd2), with associated GO terms “killing of cells of another organism” and “defense response to fungus”. Additionally, a seed linoleate 9S-lipoxygenase-3-like gene was also among the genes exclusively upregulated in ‘PI180693’, as well as Psat7g091800.1, a putative receptor-like kinase (RLK) involved in plant defense (Table 2, Table S5). Psat7g091800.1 segregated with the foliar wilt Fwt-Ps7.1 major-effect QTL on chromosome 7 that was detected in greenhouse experiments, as well as the minor- to moderate-effect QTL for ARR tolerance Ae_MRCD1_Ps-7.1, detected in field experiments [27]. Out of these four DEGs specifically upregulated in ‘PI180693’, Psat7g091800.1 was chosen for further analysis.

**Table 2:** Differentially expressed genes in ‘Linnea’ and ‘PI180693’ at 48 hpi previously described to be segregating with partial resistance to Aphanomyces root rot

Gene id	Putative biological role^a^	Linnea UK16	Linnea SE51	PI180693 UK16	PI180693 SE51
Psat1g105280.1	Methylsalicylate degradation	3.91	-0.30	2.18	1.53
Psat1g156920.1	Abscisic acid-activated signaling pathway	2.03	0.00	0.33	0.17
Psat2g013520.1	Signal transduction	n.s	n.s	3.13	1.15
Psat2g056400.1	Unknown	-1.26	-0.61	-0.23	2.23
Psat2g132720.1	Regulation of defense response	-1.53	0.12	-0.25	-0.22
Psat2g133040.1	Enhance wheat FHB resistance	5.19	0.40	3.92	0.66
Psat3g072480.1	Regulation of defense response	-1.56	-0.15	-0.09	0.35
Psat3g126560.1	Unknown	3.49	0.25	1.67	-0.84
Psat3g126600.1	Signal transduction	3.51	0.53	1.67	-0.85
Psat3g156760.1	Unknown	-1.32	-0.13	-0.91	0.74
Psat4g025040.1	Abscisic acid-activated signaling pathway	1.38	0.07	0.01	-2.58
Psat4g136120.1	Enhance wheat FHB resistance	2.89	0.43	1.68	0.64
Psat4g138760.1	Plant stress tolerance	-1.77	-0.20	-0.22	-0.43
Psat4g140440.1	Regulation of defense response	2.91	0.82	2.45	1.28
Psat4g152600.1	Unknown	4.68	0.51	2.79	1.46
Psat4g180200.1	Defense response	-1.12	0.29	-0.42	0.15
Psat4g184760.1	Jasmonic acid biosynthesis	-3.64	-0.22	-1.13	0.73
Psat4g185080.1	Jasmonic acid biosynthesis	-6.73	-0.59	-2.57	-1.20
Psat4g186560.1	Defense response	-3.62	0.35	-1.74	1.11
Psat4g188320.1	Unknown	-1.56	0.23	-0.62	0.79
Psat4g201520.1	Unknown	6.75	1.32	4.44	1.39
Psat4g201600.1	Unknown	7.61	1.75	4.90	1.00
Psat5g066680.1	Unknown	1.31	0.52	0.48	1.07
Psat5g242440.1	Defense response	3.53	1.27	3.44	2.76
Psat5g242600.1	Defense response	2.90	-2.40	4.62	8.26
Psat5g289880.1	Jasmonic acid biosynthesis	-1.51	-0.11	-0.01	5.41
Psat5g291320.1	Jasmonic acid biosynthesis	-0.66	0.10	0.41	5.74
Psat6g011200.1	Unknown	1.75	0.21	2.52	-0.51
Psat6g042720.1	Defense response	-3.31	-0.52	-0.76	-0.51
Psat6g042840.1	Defense response	-3.19	-0.11	-1.52	-0.86
Psat6g043800.1	Defense response	1.68	2.36	n.s	n.s
Psat6g144560.1	Plant defense	-2.13	-0.01	-0.43	-0.48
Psat6g146320.1	Defense against ecrotrophic fungi and abiotic stress tolerance	-1.11	-0.01	-0.22	-0.07
Psat6g203640.1	Plant defense	3.07	0.08	1.76	0.11
Psat6g207920.1	Biotic, abiotic stress, plant growth	4.32	2.09	2.33	0.50
Psat7g067680.1	Unknown	-2.12	-1.00	0.07	1.09
Psat7g083880.1	Regulating defense response	4.35	0.98	2.80	0.59
Psat7g091800.1	Plant defense	0.15	0.41	1.26	-0.78
Psat7g094400.1	Plant defense	1.51	0.12	0.56	0.09

^a^Gene function, biological processes/pathways from Wu et al. 2021 & 2022, differential gene expression compared to mock treatments, absolute values of log2FC > 1, adjusted *p*-value < 0.05, *n.s*. = not significant

### The receptor-like kinase Psat7g091800.1 is polymorphic between the resistant and susceptible pea genotypes

**Fig. 4:**
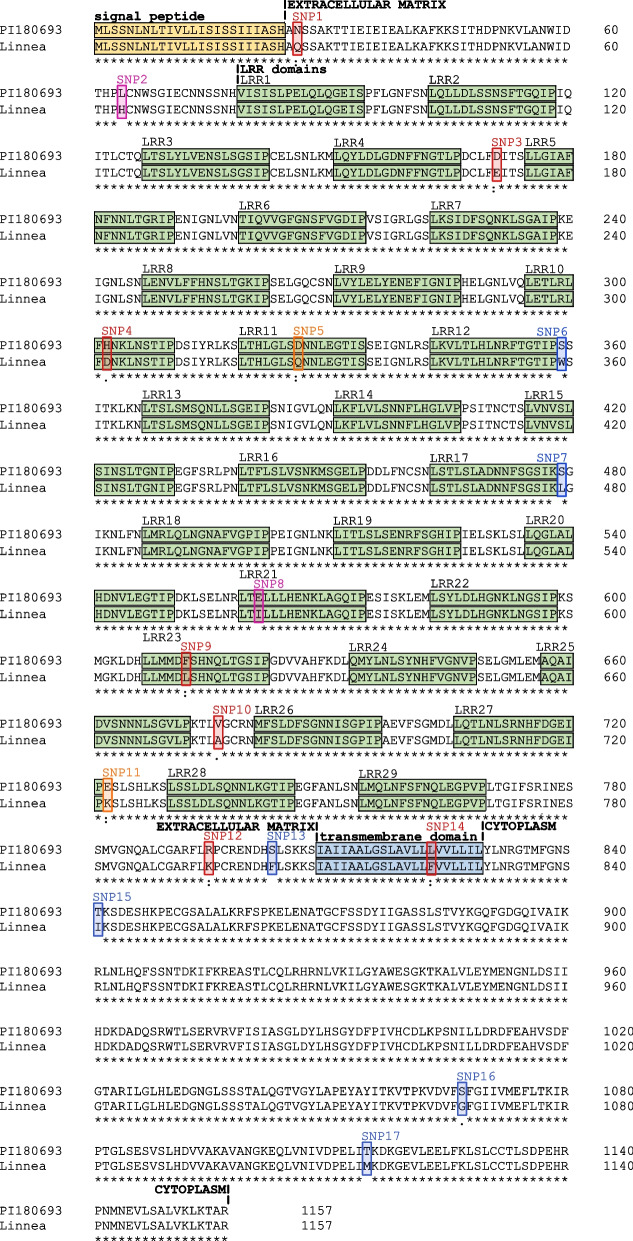
Protein alignment of the leucine-rich repeat receptor-like kinase (LRR-RLK) encoded by gene Psat7g091800.1 in pea genotypes ‘Linnea’ and ‘PI180693’. The protein sequence of ‘Linnea’ is identical to the reference sequence of the cultivar ‘Caméor’. Amino acid substitutions altering polarity are highlighted in pink, changes in charge in orange and changes in both in blue. Nonsynonymous SNPs resulting in either change are marked in red

Psat7g091800.1 was located on chromosome 7 in the genome of the pea reference cultivar Caméor with exact coordinates chr7LG7:153,683,713–153,687,363. The annotation of gene Psat7g091800.1 is therefore 3650 bp whereas in our data, reads aligned starting from the second start codon, indicating that the actual full gene length was 3645 bp in ‘Linnea’ and ‘PI180693’. Moreover, in the existing annotation, the gene has a long 3’-UTR region that encompasses a neighboring gene, but our read alignment did not support this and therefore the gene annotation was corrected to end at base 153,687,363 of chromosome 7 (Figure S3). The alternative start codon and the shorter 3’-UTR sequence was supported by a de-novo assembly of the transcript based on our RNA sequencing data. The gene had an exon–intron-exon structure with a 131 bp long intron, which had a 24 bp deletion in ‘PI180693’. The Sanger sequences from genomic DNA of ‘Linnea’ and ‘PI180693’, together with the RNAseq data revealed 39 single nucleotide polymorphisms (SNPs), with 17 leading to non-synonymous mutations (Fig. 4, Table S7). No polymorphisms were found between ‘Linnea’ and the reference sequence of the cultivar ‘Caméor’. Psat7g091800.1 was predicted to have a 24 amino acid (aa) long signal peptide, a 21 aa long transmembrane domain and 29 LRRs. Four of the SNPs between ‘Linnea’ and ‘PI180693’ were located in LRR10, LRR11, LRR21 and LRR23, and one SNP in the transmembrane domain. Eight SNPs resulted in aa changes associated with changes in polarity in the protein (Fig. 4). Domain searches in Psat7g091800.1 using Interproscan revealed similarities to the FLAGELLIN SENSING 2 (FLS2)-like domain, previously characterized as a LRR transmembrane receptor kinase crucial for flagellin perception in *Arabidopsis thaliana* [36]. Phylogenetic analyses using the entire Psat7g091800.1 protein sequence from ‘PI180693’, as well as the FLS2-encoding domain only, in comparison to homologs in other crop species showed that evolution of the Psat7g091800.1 protein followed the evolution of the analyzed species. This is also reflected in the Psat7g091800.1 protein sequence sharing > 70% sequence identity with all other legume species. In fact, the ‘PI180693’ Psat7g091800.1 protein sequence shared only 53.4% sequence identity with the *A. thaliana* homolog and thus encoded a LRR-RLK protein phylogenetically distinct from FLS2 (Figure S4).

## Discussion

Our study presented a reliable experimental setup for pea transcriptomics experiments for assessing early stages of *A. euteiches* infection. The infection controls in every biological replicate, as well as the observed exponential increase in reads mapped to the *A. euteiches* reference genome, indicated an increase in pathogen biomass during the infection process. Moreover, the estimated *A. euteiches* biomass increase correlated to an increase in number of differentially expressed genes (DEGs) over time points and higher numbers of DEGs upon infection with UK16 than SE51. The three root harvesting time points have previously been sampled in a study on the quantification of DNA and RNA transcripts of *P. pisi*, another root-rot causing oomycete pathogen of pea. During infection, *P. pisi* DNA was detectable by qPCR from 2 hpi and peaking at 48 hpi when hyphae had been accumulating in root tissue [37]. Not only were more DEGs counted in the susceptible genotype but also more defense-related genes such as predicted receptor-like kinases than in the resistant ‘PI180693’. It has previously been shown how resistance in ‘PI180693’ inhibited the production rate of oospores on infected pea root tips, associated with slower lesion development and pathogen growth than in susceptible pea lines [38]. Lavaud et al. [21] used ‘PI180693’ as a donor line for the development of Near Isogenic Lines (NILs) in their experiments and showed how root colonization and symptom appearance by *A. euteiches* can be slowed down by single or multiple resistance quantitative trait loci (QTL). Reduced oospore colonization in resistant compared to susceptible lines was also observed in *M. truncatula* infections with *A. euteiches* [9]. The host-specific immune response due to quantitative levels of resistance in ‘Linnea’ and ‘PI180693’ was further reflected in the clear separation of samples according to pea genotype in PCA analysis, as well as the lack of clustering according to virulence levels of *A. euteiches* strains. In summary, inoculation with *A. euteiches* resulted in different transcriptomic responses between the two pea genotypes that may relate to differences in disease resistance.

We observed only a few DEGs at 6 hpi and were not able to observe a clear pattern of gene regulation between pea genotypes or in response to varying *A. euteiches* virulence levels. However, among downregulated genes in the susceptible pea genotype ‘Linnea’, we found three seed linoleate 9S-lipoxygenase-3-like genes that were associated with partial resistance to ARR [27]. At 20 hpi, two of these seed linoleate 9S-lipoxygenase-3-like genes (Psat5g289880.1 and Psat5g291320.1) were among the most downregulated genes in ‘PI180693’, indicating a non-host specific downregulation of these genes. Interestingly, at 48 hpi, Psat5g289880.1 and Psat5g291320.1 were among the most highly upregulated genes in the resistant pea genotype, ‘PI180693’, in interactions with the less virulent *A. euteiches* strain. One of them, Psat5g291320.1, was previously shown to segregate with partial resistance to Aphanomyces root rot (ARR) [26]. 9S-lipoxygenases oxygenate linoleic and linolenic acid in interactions with pathogens, generating various oxylipins including precursors to the hormone jasmonic acid (JA) [39, 40]. JA signaling has been associated with plant defense to necrotrophic pathogens [41, 42]. *Aphanomyces euteiches* undergoes a shift from a biotrophic to a necrotrophic lifestyle in later stages of infection [10]. In soybean roots, higher levels of JA were observed at later time points after inoculation with the oomycete *Phytophthora sojae* [43]. Furthermore, it was recently reported that soybean cultivars with different resistance levels to *P. sojae* accumulate different levels of oxylipins. In fact, the partially resistant cultivar generally increased the production of oxylipins upon attack, suggesting that production of oxylipins may be a critical component of the defense strategies used in resistant cultivars against *P. sojae* [44]. In this context and in light of the differentially expressed lipoxygenases it would be interesting to determine the accumulation of oxylipins in ‘PI180693’ during *A. euteiches* infection.

The putative disease resistance proteins Pi176 and Pi49 are highly similar in sequence and both genes were specifically upregulated in ‘PI180693’ at 20 hpi and were originally isolated as cDNAs in pea that showed a large induction of expression in tissue responding to infections with *Fusarium solani* [45, 46]. Pi49 was assigned to class 10 (PR10)-like abscisic acid (ABA)-responsive proteins and an ortholog was found to be significantly induced in *M. truncatula* upon infection with *A. euteiches* at 6 hpi. However, the induction correlated with *A. euteiches* infection development rather than host resistance responses [47–49].

In our experiment, we also saw a significant and specific upregulation of a 9-cis-epoxycarotenoid dioxygenase gene in ‘PI180693’ seedlings interacting with the low virulent strain. This gene encodes a key enzyme involved in the biosynthesis of ABA suggesting that ‘PI180693’ seedlings accumulate ABA. Liang and Harris [50] described the role of ABA in the induction of lateral root formation in all nodulating and non-nodulating legume species. Low doses of ABA and ethylene can stimulate lateral root formation in legumes. However, in this study, the pea seedlings were not grown longer than 48 hpi, which was too early to compare lateral root formation between the pea genotypes. From previous experiments with the same pea genotypes, we know that the resistant ‘PI180693’ is able to develop a bigger root system with more lateral roots upon *A. euteiches* infection, compared to ‘Linnea’ [17] and increased root volume and architecture has been correlated with resistance to ARR in pea [51]. Higher numbers of secondary roots were also observed in the *M. truncatula* line A17, resistant to ARR, when compared with more susceptible lines [9]. From our gene expression data, it is unclear which role ABA plays in the defense against *A. euteiches* and/or lateral root formation. In summary, we have evidence for differential regulation of ABA-responsive and biosynthesis genes between pea genotypes and hypothesize that the ABA signaling might be important for resistance in ‘PI180693’.

The transcription factors myeloblastosis (MYB)14 and MYB15 were among the most strongly upregulated genes in both pea genotypes at 48 hpi. These genes belong to subgroup 2 of the MYB transcription factors that control phenylpropanoid metabolism. Members of this group are involved in stilbene biosynthesis in *Vitis vinifera* (VvMYB14 and 15), and isoflavonoid biosynthesis in *Lotus japonicus* in response to biotic and abiotic stress [52, 53]. Interestingly, we found *myb14* and *myb15* and other MYB-like transcription factors almost exclusively upregulated upon infection with the more virulent *A. euteiches* strain UK16. The *rax3* MYB transcription factor gene, which was strongly upregulated in all interactions except between ‘PI180693’ and UK16, is an ortholog of the *A. thaliana MYB84* gene. The *A. thaliana* *myb84* is a member of a network of MYB transcription factors that interact with ABA signaling to control suberin biosynthesis in root development and stress responses [54]. Another transcription factor with a similar expression pattern in this study is the pea ortholog of the *A. thaliana wrky18* gene. *wrky18* is quickly induced by ABA to inhibit root growth [55]. The ortholog of *wrky40*, an antagonist to *wrky18* [55], was significantly upregulated at 20 hpi in the interaction between ‘Linnea’ and UK16. This is further supporting a role of ABA signaling and root growth in the interaction between pea and *A. euteiches.*

By cross-referencing our DEG data set with genes located in genomic regions shown to segregate with ARR resistance in pea [26, 27], we arrived at 39 candidate disease resistance genes. The susceptible and resistant pea genotypes shared a higher proportion of commonly upregulated than downregulated genes and we found no genes specifically downregulated in ‘PI180693’ segregating with partial resistance to ARR. The four specifically upregulated DEGs in the resistant pea genotype were of special interest as they might reflect the genotype-dependent resistant phenotype. The gene Psat7g091800.1 presented an interesting candidate for further Sanger sequencing as it segregated with the ARR tolerance Ae_MRCD1_Ps-7.2 QTL on pea chromosome 7 [27] and displayed a classical nucleotide-binding domain leucine-rich repeat (NLR) immune receptor structure. NLRs account for the largest family of plant resistance genes, and act by recognizing pathogen effectors delivered into the host and subsequently induce host cell death and resistance responses [56–58]. The Psat7g091800.1 allele in ‘PI180693’ displayed a number of potentially adaptive amino acid (aa) substitutions compared to the allele in ‘Linnea’, as well as the pea reference genome from pea genotype ‘Caméor’ [18]. This is likely due to the fact that ‘Caméor’ as a bred cultivar (released in 1973) had been undergoing similar genetic selection steps as other commercial cultivars, resulting in a more similar genome than the old landrace ‘PI180693’ [15]. As four nonsynonymous single nucleotide polymorphisms (SNPs) were located within LRRs, the functionality of the immune receptor during pathogen defense in ‘Linnea’ might be compromised. However, to make further assumptions about the functionality of the immune receptor and its use in pea breeding, functional validation is required. The pattern recognition receptor (PRR) FLS2 was originally described in *A. thaliana* as being involved in the perception of the microbe-associated molecular pattern (MAMP) flagellin [36, 59]. In our analysis, the FLS2-like domain in Psat7g091800.1 showed to share only 58.2% sequence identity with the FLS2-encoding domains in *A. thaliana.* Moreover, phylogenies based on sequence homology reflected taxonomic differences between plant families rather than unique FLS2-like domains conserved in other plant species. In summary, Psat7g091800.1 encodes a putative NLR immune receptor that constitutes a candidate ARR disease resistance protein.

## Conclusion

In conclusion, our work showed how transcriptomic data was successfully combined with available data on ARR resistance QTL to identify candidate disease resistance genes in pea. We gained insights on the transcriptomic immune response in pea to ARR, which has shown to be time-dependent. Differences in differential gene expression were clear between the resistant and susceptible pea genotype but much more subtle between *A. euteiches* virulence levels, representing a non-strain specific quantitative disease resistance mechanism in pea towards ARR. Furthermore, the 39 candidate disease resistance genes presented in this study pose a valuable resource for future marker-assisted selection in pea breeding programs. We were also able to identify a polymorphic, putative NLR immune receptor gene specifically induced in the partially resistant ‘PI180693’ pea genotype. Functional validation of this gene is required to assess its exact function in ARR disease resistance and its usefulness in pea resistance breeding programs.

## Materials and methods

### *Aphanomyces euteiches* cultivation and zoospore induction

For the *A. euteiches* infections in this study, we used strain SE51, from southern Sweden, and UK16 from the United Kingdom. Strain SE51 has been used for many years in Swedish pea breeding programs as a reference for low pathogen virulence. On the contrary, strain UK16 has been shown to be highly virulent on both ‘Linnea’ and ‘PI180693’ in growth chamber trials [17]. Both strains were included in a previous study on the genetic diversity of *A. euteiches* in Europe and were found to cluster together in a genetically similar, central European group [16]. Strains SE51 and UK16 were grown on corn meal agar (CMA; BD Biosciences) plates at 20 °C in the dark for two weeks. Inoculum preparation was performed following the protocol by Hosseini et al. [37], with few modifications. Five agar plugs (7 mm diameter) were used as inoculum in 200 ml V8 vegetable juice medium liquid cultures and grown in the dark at 25 °C for five days. For medium preparation, the vegetable juice (Eckes-Granini Group) was filtered through a miracloth (Merck Millipore) and diluted with sterilized water to a 20% solution, following addition of 0.3 g/L CaCO_3_ and autoclaving. To induce zoospore production, the V8 medium was decanted, and the cultures were washed once with autoclaved river water (Fyrisån, Uppsala), followed by a three-hour incubation period in new river water at 25 °C in the dark for two days. The zoospore concentration was measured using a hemacytometer and adjusted with autoclaved tap water to 5 × 10^4^ spores/ml.

### Pea material and germination

In this experiment, the commercial pea cultivar ‘Linnea’ was used as a susceptible, and the partially resistant line ‘PI180693’ as a resistant genotype for *A. euteiches* infections [17]. Seeds were surface sterilized by several washing steps using 1% sodium hypochlorite and 70% ethanol as described in Kälin et al. [16] prior to pre-germination on 0.8% water agar plates at 20 °C for three days in the dark.

### Experimental setup, inoculation and harvest

The experiment was conducted in a balanced replicated design with both pea genotypes represented in every of the five biological replicates (200 μl pipette tip boxes) as shown in Figure S1. Inoculation with *A. euteiches* strains SE51 and UK16 was performed simultaneously by placing the racks of the pipette boxes with protruding roots in respective zoospore solution (concentration 5 × 10^4^ spores/ml) for 30 s, before transferring to new pipette boxes with autoclaved tap water. The replicates were kept open in a growth cabinet (20℃, 70–80% humidity, 12 h light, 12 h dark, 150 μmol per m^2^/s) until sampling. Pea roots were sampled at 6, 20 and 48 h post inoculation (hpi), with two roots per genotype, treatment and biological replicate. The seedling development stages ranged from seedlings with only radicle and plumule at 6 hpi to the formation of scale leaves at 48 hpi (BBCH identification keys 07 to 09–10). The roots were cut five mm from the proximal end and immediately frozen in liquid nitrogen and stored at -70 °C.

### RNA extraction, quality control and sequencing

Three glass beads (2 mm diameter) were added to each 2 ml screw cap tube containing two frozen pea roots and extraction buffer. A Precellys 24 Tissue Homogenizer (Bertin Technologies) was used at 5500 rpm for 2 × 30 s. RNA was extracted using the Spectrum Plant Total RNA Kit (Sigma-Aldrich), following protocol A as described in the manufacturer’s protocol. In brief, homogenized samples were incubated at 56 °C for 3 min and centrifuged at 17,000 × g (Heraeus Pico 17 Microcentrifuge, Thermo Scientific). The lysate supernatant was collected and filtered through a column by centrifugation at 17,000 × g. The clarified lysate was collected in a clean tube, added with 500 μl of the binding solution and mixed immediately. The mix was transferred into a binding column and centrifuged at 17,000 × g for 1 min. After washing the column with wash solution, total RNA was eluted with two elution steps following procedures described by the manufacturers (Sigma-Aldrich). The column and solutions used in RNA extractions were provided in the kit. Extracted RNA was then diluted in nuclease-free water and measured with RNA Qubit RNA High Sensitivity (Thermo Scientific). Approximately 1000 ng RNA were used for subsequent DNase treatment in 10 µl reactions using DNase I (Thermo Scientific) with additional RNase inhibitor. DNase-treated RNA was run on an RNA Nano Chip on a 2100 Bioanalyzer System (Agilent Technologies) for quality assessment. Three biological replicates were chosen for sequencing and submitted to NGI sequencing facility (SciLifeLab, Uppsala) for library preparation for a total of 54 libraries (TruSeq Stranded Total RNA kit with Ribo-Zero Plant) and sequencing on a NovaSeq6000 S4 lane, 150 bp paired-end.

### Transcriptomic analysis

Sequencing adapters removal and quality trimming was performed using Bbduk v. 38.90 [60] with the following parameters:

$$\text{ktrim}=\mathrm r\;\mathrm k=23\;\mathrm{mink}=11\;\mathrm{hdist}=1\;\mathrm{tpe}\;\mathrm{tbo}\;\mathrm{qtrim}=\mathrm r\;\mathrm{trimq}=10.$$

MultiQC v. 1.12 [61] was then used for checking the quality of the cleaned reads. To avoid mismapping, a combined genome index for the *A. euteiches* reference genome ATTCC201684 [62] and the sequenced pea genome of the French cultivar Caméor [18] was generated using STAR v. 2.7.9a [63] and the following settings:

$$-\text{sjdbOverhang}\ 100-\text{sjdbGTFfeatureExon CDS} -\text{sjdbGTFtagExonParentTranscript Parent}-\text{genomeSAindexNbases} 10.$$

The reads were mapped to the combined genome using STAR v. 2.7.9a with default parameters, and then read count tables were obtained using featureCounts v. 2.0.1 [64] with the following options:

$$-\mathrm p-\mathrm t\;\mathrm{exon}-\mathrm g\;\mathrm{Parent}-\mathrm B-\text{C.}$$

### Differential gene expression analysis and visualization

The R package DESeq2 (ver. 1.32.0) was used with default parameters for differential gene expression analysis and principal component analysis (PCA) plots were generated with regularized log transformation. Contrasts were set comparing infection with *A. euteiches* strains, time points and genotypes to the same conditions, but mock treated. Genes with less than ten total read counts in a single contrast were dismissed from the analysis and genes were considered differentially expressed with log2FC values >  < 1 with FDR adjusted *p*-values of < 0.05. A list of genes segregating with partial resistance to ARR in pea as described in Wu et al. [26, 27] was used to further filter genes of interest. The online platform *Bioinformatics & Evolutionary Genomics* (https://bioinformatics.psb.ugent.be/links/credits) was used to illustrate DEGs in Venn diagrams. BAM files were loaded into the integrative genome viewer IGV (version 2. 12. 3 03) [65] for visualization of gene expression.

### Gene ontology enrichment analysis and homology searches

The public annotation of the pea genome was downloaded from https://urgi.versailles.inra.fr/download/pea/ and gene ontology (GO) enrichment analysis was done through Fisher’s exact tests with FDR-adjusted *p*-value of 0.05 as threshold. The Fisher tests were run using the agriGO online service [66] for simple enrichment analysis, and the enriched GOs were visualized using REVIGO with redundancy filtering [67]. The functional annotation available on the pea database was complemented with InterProScan (v. 5.48) and BLASTp analysis against the NCBI non redundant protein database, using a minimum ID of 60% and minimum query coverage of 80%.

### Sequencing, SNP calling and analysis of Psat7g091800.1

Genomic DNA was extracted from roots of ‘Linnea’ and ‘PI180693’ using the DNeasy Plant Mini Kit (Qiagen) following the manufacturer’s protocol. Primers were designed based on the reference sequences using DNASTAR (v. 17.2.1.61) software. PCR amplification of the Psat7g091800.1 gene were run on a Veriti™ 96 well Thermal Cycler (Applied Biosystems) using respective primers (Table S1). Each reaction contained 25 ng of template DNA and was conducted following the PCR protocol for Phusion Polymerase with 0.5 µM primer concentration in a total of 25 μl reaction volume. The initial denaturation was at 98 °C for 30 s, followed by 32 cycles at 98 °C for 10 s respective annealing temperature for 20 s and extension at 72 °C for 90 s. The concentrations of PCR products were determined with absorbance measurements on a NanoDrop 1000 Spectrophotometer and electrophoresis in 1.5% agarose gels was performed for verification of fragment size. The PCR products were purified using AMPure XP reagent (Beckman Coulter) and concentrations were adjusted to 50 ng/µl for each product prior to submitting to Macrogen Europe B.V. (Amsterdam, Netherlands) for Sanger sequencing. Contig assemblies were done using SeqMan Ultra (v. 17.2.1) and alignments were done in MEGA-X v. 10.0.5 [68] where single nucleotide polymorphism (SNP) calling was done manually. PhytoLRR [69], SignalP [70] and DeepTMHMM [71] were used for prediction of LRRs and functional domains. The variant effect predictor by EnsemblPlants (release 109) [72] was used to assess consequence types of SNPs, and the mapping of RNA reads on the gene were visualized through the Integrative Genomics Viewer (v. 2.15.4). To obtain a de-novo transcript sequence of the gene, the command “samtools faidx” [73] was used to isolate, from the bam files generated with STAR, the reads mapping within 2000 bp of the reported location of Psat7g091800.1. Said reads were then corrected using Rcorrector [74] with default parameters, the unfixable reads were removed, and the remaining ones were assembled through Trinity v. 2.11.0 [75] with default parameters.

### Orthologs and phylogenetic analyses

The predicted Psat7g091800.1 protein sequence was compared against the NCBI protein database using the psi-BLAST algorithm [76] in a selection of representative cultivated organisms of different plant families, including pea, chickpea, soybean, white clover, *M. truncatula*, potato, tomato, wild cherry, rapeseed, *A. thaliana*, cucumber and melon. The protein sequence of the best hit for every species was used for a sequence alignment with the multiple sequence alignment program MAFFT (v. 7.453) [77] and the L-INS-I accuracy-oriented method with following options:

$$-\text{localpair }-\text{maxiterate }1000.$$

Phylogenetic trees were computed using IQ-TREE (v. 2.1.3) [78] using the ModelFinder option [79] with following settings:

$$-\text{seed }17-\text{st AA }-\text{m MFP }-\text{b }1000-\text{safe }-\mathrm T\;1.$$

For the alignments of the entire protein and the FLS2-encoding domains, the best model according to Bayesian information criterion (BIC) scores was Q.plant + G4 for the construction of a maximum likelihood tree. Condensed trees were computed in MEGA-X v. 10.0.5 [68] with a bootstrap cutoff value of 70% (Figure S4).

### Supplementary Information

**Additonal file 1: Figure S1.**

**Additonal file 2: Figure S2.**

**Additonal file 3: Figure S3.**

**Additonal file 4: Figure S4.**

**Additonal file 5: Table S1.** Primer sequences, amplicon size and annealing temperatures for gDNA amplification of Psat7g091800.1.

**Additonal file 6: Table S2.** Read counts on Aphanomyces euteiches and Pisum sativum.

**Additonal file 7: Table S3.** Number of differentially expressed genes in every condition compared to the mock treatments.

**Additonal file 8: Table S4.** Significant results of the DESeq2 analysis. All combinations of pea genotype, strain and time point were compared to the mock inoculation in the same condition.

**Additonal file 9: Table S5.** DEGs at 6 hpi, 20 hpi, 48hpi and resistance DEGs.

**Additonal file 10: Table S6.** Enriched GO terms of 48 hpi DEGs.

**Additonal file 11: Table S7.**  Positions of single nucleotide polymorphisms, including changes in amino acids in the gene Psat7g091800.1.

## Data Availability

The transcriptome data is available on the European Nucleotide Archive (ENA, https://www.ebi.ac.uk/ena/browser/search) under Bioproject “PRJEB66187”.

## Abbreviations

ARR: Aphanomyces root rot
QTL: Quantitative trait locus/loci
BSR-seq: Bulked segregant RNA-seq
DEGs: Differentially expressed genes
PCA: Principal component analysis
GO: Gene ontology
JA: Jasmonic acid
ABA: Abscisic acid
TF: Transcription factor
MYB: Myeloblastosis
LRR: Leucine-rich repeat
MLP: Major latex protein
RUN1: *Uncinula necator* 1
Psd2: *P. sativum* Defensin 2
RLK: Receptor-like kinase
SNP: Single nucleotide polymorphisms
aa: Amino acid
FLS2: FLAGELLIN SENSING 2
NLR: Nucleotide-binding domain leucine-rich repeat
PR: Pattern recognition receptor
MAMP: Microbe-associated molecular pattern
BLAST: Basic local alignment search tool
