# Supplementary material for: Transcriptomic analysis identifies candidate genes for Aphanomyces root rot disease resistance in pea
Source: BMC Plant Biol. 2024 Feb 28;24:144. doi: 10.1186/s12870-024-04817-y (PMC10900555; doi:10.1186/s12870-024-04817-y)
Supplement: Supplementary file 1 — Additonal file 1: Figure S1. [file 12870_2024_4817_MOESM1_ESM.pdf]

Figure S1

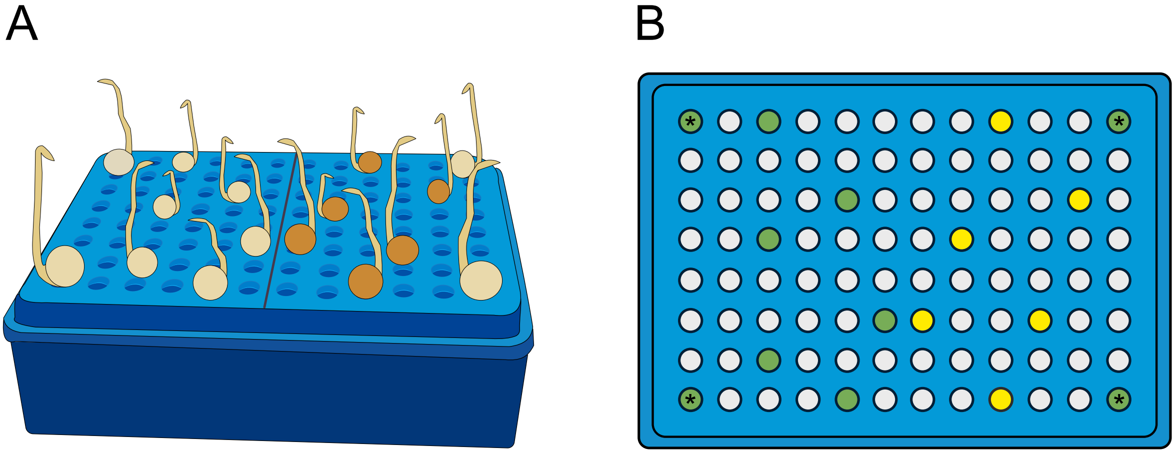

Figure S1. Experimental setup showing a three-dimensional representation of the water infection system using 200µl filter tip pipette boxes as biological replicates (A) and the top view of the setup with pea genotype 'Linnea' (green) and 'PI180693' (yellow) seedling distribution (B). Non-inoculated controls in each corner are indicated with an asterisk.
