# Supplementary material for: Transcriptomic analysis identifies candidate genes for Aphanomyces root rot disease resistance in pea
Source: BMC Plant Biol. 2024 Feb 28;24:144. doi: 10.1186/s12870-024-04817-y (PMC10900555; doi:10.1186/s12870-024-04817-y)
Supplement: Supplementary file 2 — Additonal file 2: Figure S2. [file 12870_2024_4817_MOESM2_ESM.pdf]

Figure S2

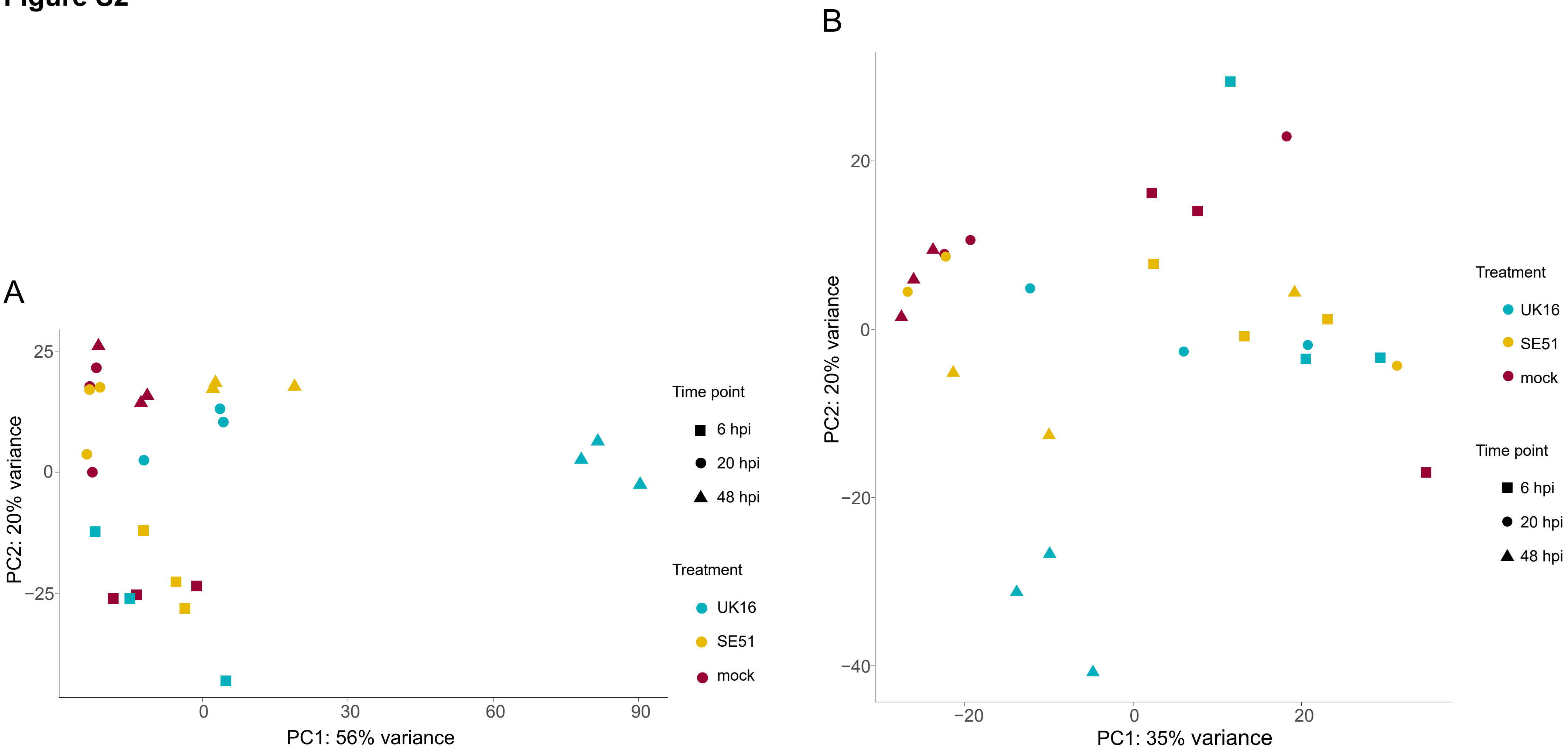

Figure S2. Principal component analyses (PCAs) split by pea genotypes 'Linnea' (A) and 'PI180693' (B). Data points of three biological replicates for every *A. euteiches* treatment (highly virulent UK16 and lowly virulent SE51) and the mock control, as well as for the root harvesting time points (shapes) are shown.
