# Supplementary material for: Transcriptomic analysis identifies candidate genes for Aphanomyces root rot disease resistance in pea
Source: BMC Plant Biol. 2024 Feb 28;24:144. doi: 10.1186/s12870-024-04817-y (PMC10900555; doi:10.1186/s12870-024-04817-y)
Supplement: Supplementary file 3 — Additonal file 3: Figure S3. [file 12870_2024_4817_MOESM3_ESM.pdf]

Figure S3

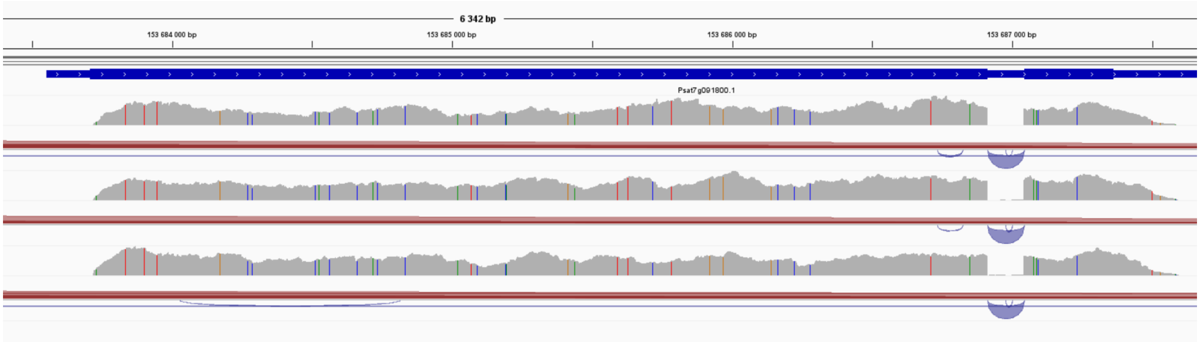

Figure S3. Reads of the three biological replicates of 'PI180693' infected with the highly virulent *A. euteiches* strain UK16 at 48 hpi mapping on gene Psat7g091800.1. Colored bars show SNPs compared to the reference gene annotation from https://urgi.versailles.inra.fr/download/pea/.
