# Supplementary material for: Transcriptomic analysis identifies candidate genes for Aphanomyces root rot disease resistance in pea
Source: BMC Plant Biol. 2024 Feb 28;24:144. doi: 10.1186/s12870-024-04817-y (PMC10900555; doi:10.1186/s12870-024-04817-y)
Supplement: Supplementary file 4 — Additonal file 4: Figure S4. [file 12870_2024_4817_MOESM4_ESM.pdf]

Figure S4

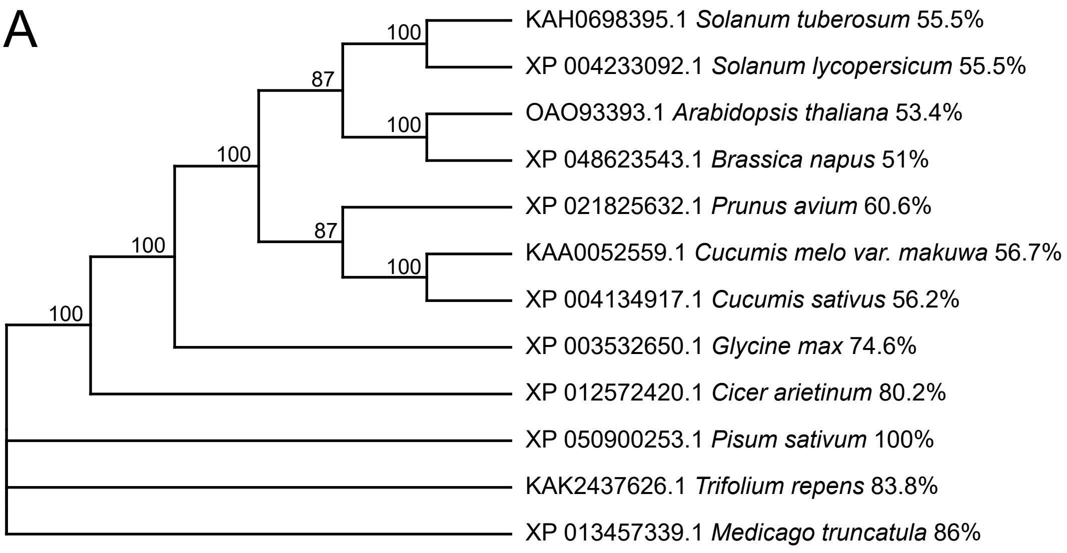

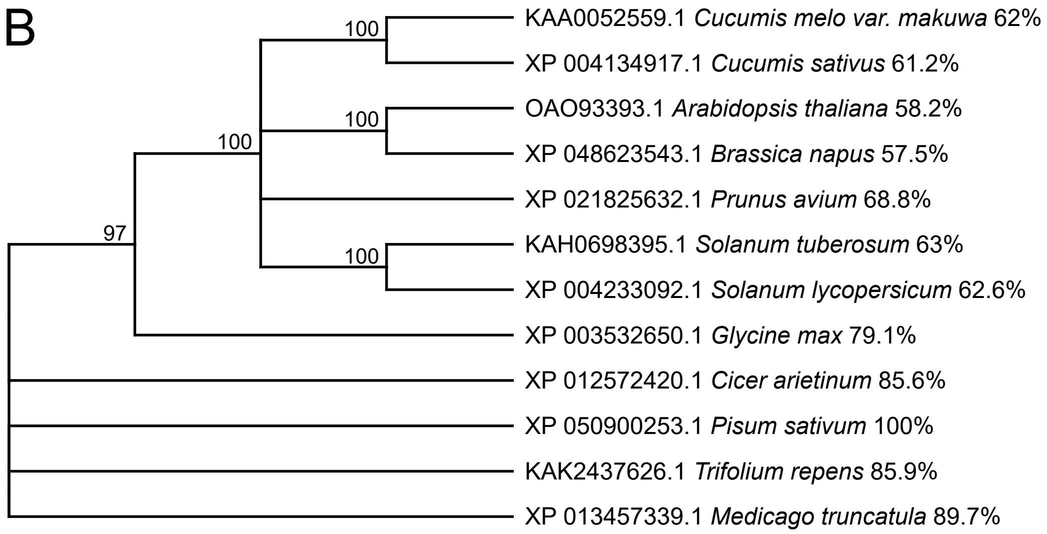

Figure S4. Phylogenies of the entire protein sequence of Psat7g091800.1 (XP 050900253.1) from 'PI180693' (A) and FLS2-like domain encoding sequence parts (B) reflect taxonomic distance of represented plant families. Homologs in other plant families have been identified with BLAST searches in the NCBI protein database, selected for best hits and maximum likelihood trees were constructed using IQ-TREE. Percent identity compared to query from *P. sativum* are indicated for every accession.
