# Supplementary material for: Transcriptomic analysis identifies candidate genes for Aphanomyces root rot disease resistance in pea
Source: BMC Plant Biol. 2024 Feb 28;24:144. doi: 10.1186/s12870-024-04817-y (PMC10900555; doi:10.1186/s12870-024-04817-y)
Supplement: Supplementary file 5 — Additonal file 5: Table S1. Primer sequences, amplicon size and annealing temperatures for gDNA amplification of Psat7g091800.1. [file 12870_2024_4817_MOESM5_ESM.docx]

**Table S1**. Primer sequences, amplicon size and annealing temperatures for gDNA amplification of Psat7g091800.1

| Primer id | Primer sequence 5’ to 3’ | Fragment size [bp] | Annealing Temperature [°C] |
| --- | --- | --- | --- |
| 091800_1F | CCCCCTCTCTCAAGTCTCAAACCT | 978 | 60,7 |
| 091800_1R | CCAACAAAACTATTCCCAAACCCT |  |  |
| 091800_3F | ACCTTGTTGAGAATTCTCTCTCCG | 674 | 61,4 |
| 091800_3R | CCCGGTGAACCTATTCAAATGC |  |  |
| 091800_4F | GGTAATTTAGTTCAGTTGGAAACATTG | 726 | 57,5 |
| 091800_4R | TTTGGACAATTCGATCGGTATATG |  |  |
| 091800_5F | AGCGGTGAACTCCCTGATGAT | 831 | 61 |
| 091800_5R | GTGTGACAAGCTTTTGGGGATTT |  |  |
| 091800_6F | ATGGCACAAGCGATTGATGTTT | 805 | 59,5 |
| 091800_6R | GGCACAAAGTGCTGGCTTCTCT |  |  |
| 091800_7F | CCGGTTGTTTCAGCTCAGACTAC | 980 | 52,2 |
| 091800_R-out | TGTTTATCATTTAACATGACAGTA |  |  |
| 091800_R-out | TGTTTATCATTTAACATGACAGTA | 640 | 52,2 |
| 091800_3’F2 | TGCGAGCGGATTGGATTACTTGC |  |  |
