# Supplementary material for: Transcriptomic analysis identifies candidate genes for Aphanomyces root rot disease resistance in pea
Source: BMC Plant Biol. 2024 Feb 28;24:144. doi: 10.1186/s12870-024-04817-y (PMC10900555; doi:10.1186/s12870-024-04817-y)
Supplement: Supplementary file 7 — Additonal file 7: Table S3. Number of differentially expressed genes in every condition compared to the mock treatments. [file 12870_2024_4817_MOESM7_ESM.docx]

**Table S3.** Number of differentially expressed genes in every condition compared to the mock treatments

| *A. euteiches* strain | Time point | Linnea | PI180693 |
| --- | --- | --- | --- |
| UK16 | 6 hpi | 8 | 4 |
| UK16 | 20 hpi | 255 | 131 |
| UK16 | 48 hpi | 6023 | 1383 |
| SE51 | 6 hpi | 64 | 1 |
| SE51 | 20 hpi | 18 | 32 |
| SE51 | 48 hpi | 218 | 318 |

*hpi= hours post inoculation, differential gene expression compared to mock treatments, absolute values of log2FC > 1 and adjusted p-value < 0.05
